# The effectiveness of the “SMG” model for health-promoting lifestyles among empty nesters: a community intervention trial

**DOI:** 10.1186/s12955-019-1222-x

**Published:** 2019-11-08

**Authors:** Chichen Zhang, Xiao Zheng, Ruifang Zhu, Lihong Hou, Xiaozhao Yousef Yang, Jiao Lu, Feng Jiang, Tingzhong Yang

**Affiliations:** 1 School of Health Services Management, Southern Medical University, Guangzhou, Guangdong China; 2 Center for Health Management and Policy Research, Shanxi Medical University, Taiyuan, Shanxi China; 3 School of Medicine, Zhejiang University, Hangzhou, Zhejiang China; 4 School of Nursing, Shanxi Medical University, Taiyuan, Shanxi China; 5 Department of Political Science and Sociology, Murray State University, Murray, KY USA; 6 Library, Shanxi Medical University, Taiyuan, Shanxi China

**Keywords:** Health management, The “SMG” model, Health-promoting lifestyles, Empty nesters, Intervention

## Abstract

**Background:**

With the disintegration of the extended family in recent years, the empty-nest phenomenon is increasingly common in China and the health of empty nesters is attracting more attention. Lifestyles, accounting for 53% in determining death, play a vital role in improving the health of individuals. However, it was rarely studied in promoting the health of empty nesters. In this study, we proposed a “SMG” model in empty nesters, including the self-management, mutual management, and group management, to implement health-promoting lifestyles interventions among empty nesters to provide an effective means to improve their lifestyles and health.

**Methods:**

We conducted a prospective intervention on 350 empty nesters in three communities located in Taiyuan, China. One hundred sixty-seven empty nesters were randomly assigned to the intervention group with SMG-based health-promoting lifestyles interventions used for 7-months, and 183 were randomly assigned to the control group with no measures imposed. The Health-Promoting Lifestyle Profile (HPLP-C) was used to rate the lifestyles of empty nesters. Generalized estimation model was used to analyze the differences between the intervention and control groups over time, adjusted for education and employment.

**Results:**

After 7 months of health-promoting lifestyles intervention, HPLP-C score and each dimension score in the intervention group all increase from baseline. There were significant differences after intervention associated with time and group interaction effects in aspects of HPLP-C (mean score = 8.838, 95%CI:6.369–11.306), self-realization (mean score = 1.443, 95%CI:0.352–2.534), Health responsibility (mean score = 1.492, 95%CI:0.477–2.508), physical activity (mean score = 1.031, 95%CI:0.572–1.491), nutrition (mean = 0.827, 95%CI:0.177–1.476), interpersonal relations (mean = 2.917, 95%CI:2.365–3.469) and stress management (mean score = 0.729, 95%CI:0.131–1.327). And education is contributing to the effect of the health-promoting lifestyle intervention (Estimate:8.833, *p* < 0.001).

**Conclusions:**

SMG-based health-promoting lifestyles intervention in empty nesters effectively improved the lifestyles of empty nesters, and the outcome was affected by education. Lifestyle change requires ongoing intervention, and community service centres must be involved in implementing the “SMG” model to provide ongoing support and improve the effect of interventions among empty nesters.

**Trial registration:**

Chinese Clinical Trial ChiCTR1800015884. Registration date: 26–04-2018. Retrospectively registered.

## Background

Before the rise of the industrialized and medicalized model of the care for older adults in most developed countries, such as through the state-funded networks including Medicare and various types of senior houses, older adults used to be taken care of exclusively by close-knitted social circles. Family and kinship are a characterizing example of such primary social circles. In a traditional Chinese family, older parents co-reside with their adult children and receive care and financial assistance from them. The theory of intergenerational flow describes that this pattern of intergenerational care existed in most countries before the demographic transition [1].

However, this traditional living arrangement has been challenged by the disintegration of the extended family in recent years, making it increasingly common that few adult children are available to help older adults when needed, while the proportion of older adults living alone is increasing. These elders are considered to be “empty nesters” [2], an analogue that children are compared to birds flying away from the nest with the elderly left behind lonely. Empty nesters may be further subclassified into relative empty nesters (who live in the same city as their children but not in the same household), absolute empty nesters (whose children live abroad or in another city in China), and empty nesters with no children (no children or children have died) [3]. In 2014, one survey conducted by the China National Committee on Ageing announced that empty nesters accounted for 51.1% of the elderly in China [4]. As the empty-nest phenomenon becomes more common, the duration of time as empty nesters is significantly extended, and the percentage of empty nesters will continue to rise in China irreversibly.

Aging and empty nesting are associated with important socioeconomic consequences that may unfavourably affect the stability of a sustainable economy and society, as well as the very wellbeing of the aging populace of China. Some researchers had demonstrated that the empty nesters were particularly vulnerable to different disadvantageous situations and experienced more physical and mental problems compared to seniors living with a child [5]. Being in an empty nest had adverse impact on elders’ health [6]. A cross-sectional study in rural China (*n* = 3182) revealed that empty nesters were 1.33 times more likely to develop depressive symptoms compared with non-empty nesters [7]. The amplification of the size of empty nesters has already pressed the aging nation’s budget in health care, social security, and the pension system. Finding a way to improve the health of the empty nesters concerns the long-term wellbeing of China’s demography.

**Fig. 1:**
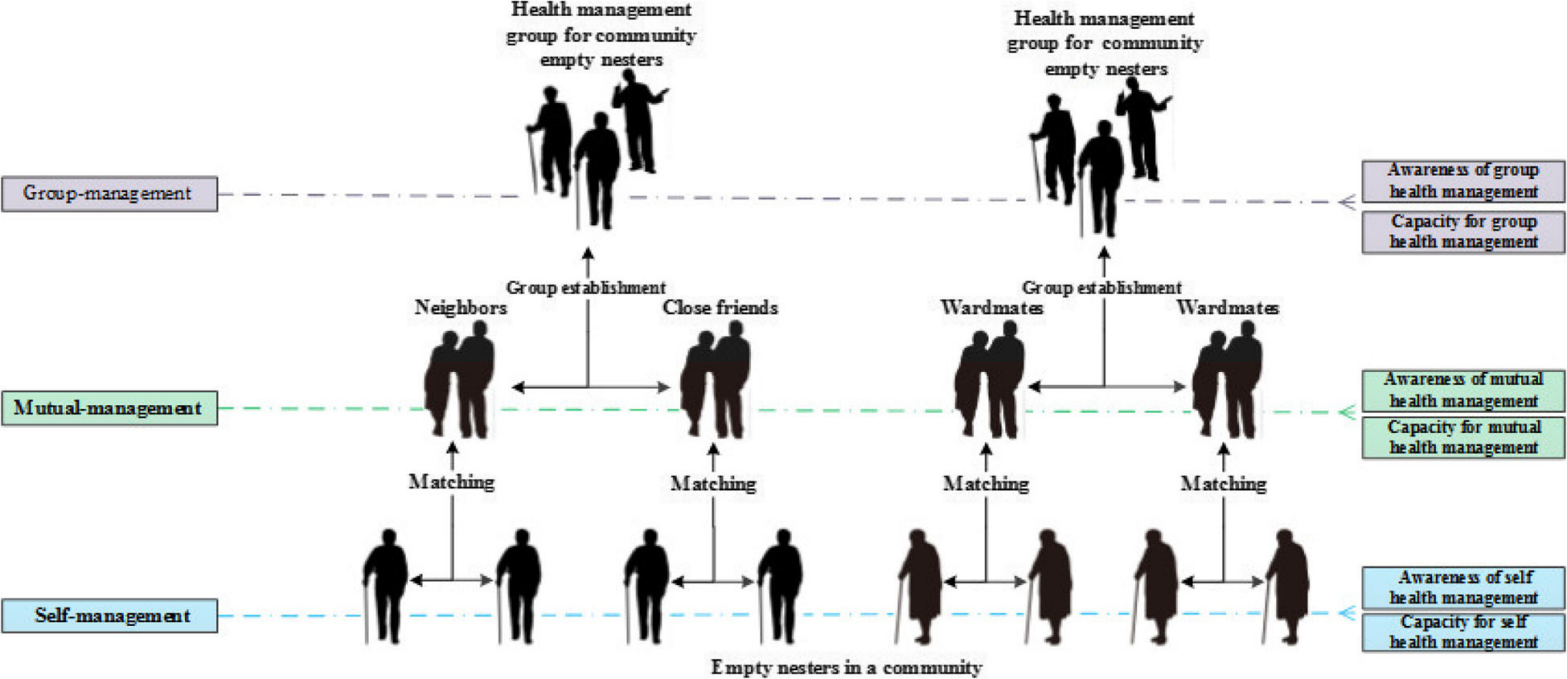
“SMG” hierarchical management roadmap. Legend: The figure was from authors’ previous paper named Study on “SMG” health management model of the empty-nest elderly based on community organization theory (theory article) [14]

Previous studies had confirmed that health promotion behaviour is one of the primary criteria for determining health and preventing disease [8, 9]. The health lifestyle theory leverages the Weberian concept of “lifestyle” to argue that certain health behaviours cluster with an individual’s rational and voluntary choice of material consumptions and social conducts that are made available by his/her life conditions [10]. For example, at-risk health behaviours such as substance use often occur together with at-risk social conducts such as aggression, creating a cluster of related behaviours known as lifestyle. Therefore, a good health lifestyle matters greatly for the physical and mental health of the empty-nest elderly. A study from WHO indicated that 53% of causes of death are related to lifestyle, 21% to environmental factors, 16% to genetic factors and 10% to healthcare service system [11]. In other populations, such as metabolic syndrome patients and the elderly, it was found that self-actualization (A need to be good, to be fully alive and to find meaning in life.), good nutrition habits and frequent physical activities were correlated with fewer depressive symptoms [12–14]. A study based in South Korea found positive correlations between the elderly’s social networks and both health-promoting behaviour and health-related quality of life [15]. An Iranian study reported that the quality of life among the elderly could be improved through health-promoting interventions targeting the maintenance and increase of physical activities, stress management, and spiritual growth [16]. Kilbourne [17] conducted a RCT in the field of health lifestyle management and proved the positive effects of an intervention named “life Goals Collaborative Care” (LGCC) compared with the controlled one. Frank [18] reported that an integrated risk reduction intervention (IRRI) including the lifestyle coaching presented an instructive effectiveness. And there are some health promotion intervention programs provide multiple interventions for older adults, since the health domains of older adults covers physical, mental, and social dimensions. The findings from a 2 years multidomain intervention of diet, exercise, cognitive training, and vascular risk monitoring versus control, suggest that a multidomain intervention could improve or maintain cognitive functioning in at-risk elderly people from the general population [19]. Clark [20] reported that a lifestyle-oriented occupational therapy intervention has beneficial effects for ethnically diverse older people recruited from a wide array of community settings. A study conducted by Chen reported that the sleep quality, depression, and health status of older adults could be improved by silver yoga exercises. Nevertheless, the previous intervention studies only tended to adopt single intervention strategy, but did not be stratified or took different interventions at different stages of the intervention procedure. Due to the weakness in knowledge, we proposed the Self-management, Mutual management, and Group management model (SMG model) of health promotion for empty nesters [21]. This model is a multi-level integrated health management model of which the core is to elevate the self-efficacy and the aim is to improve health in empty nesters. The management subject involves empty nesters themselves and relevant management staff or institutions, and the model may be implemented at home, in communities, and at community health service centres. Figure 1 [21] depicts the management roadmap based on the survey results of the health management status of empty nesters and modern health management concepts and strategies (As shown in Fig. 1: “SMG” hierarchical management roadmap).

In the “SMG” model, the whole intervention procedure was specifically divided into three stages, and the participants were paired and grouped in terms of the disease types. And the different stages and groups were treated with different intervention strategies. We hypothesized that the SMG model could effectively improve the health-promoting lifestyles of empty nesters. To test this innovative and promising model, we selected a medium size city in China as the site to implement SMG-basedhealth-promoting lifestyle intervention among empty nesters and conducted a community-based trial to quantify the intervention effect of the SMG model on health-promoting lifestyle. If the health-promoting lifestyle of empty nesters are improved, the results will provide new intervention strategies for improving the lifestyle and health of empty nesters.

## Methods

### Sample

**Fig. 2:**
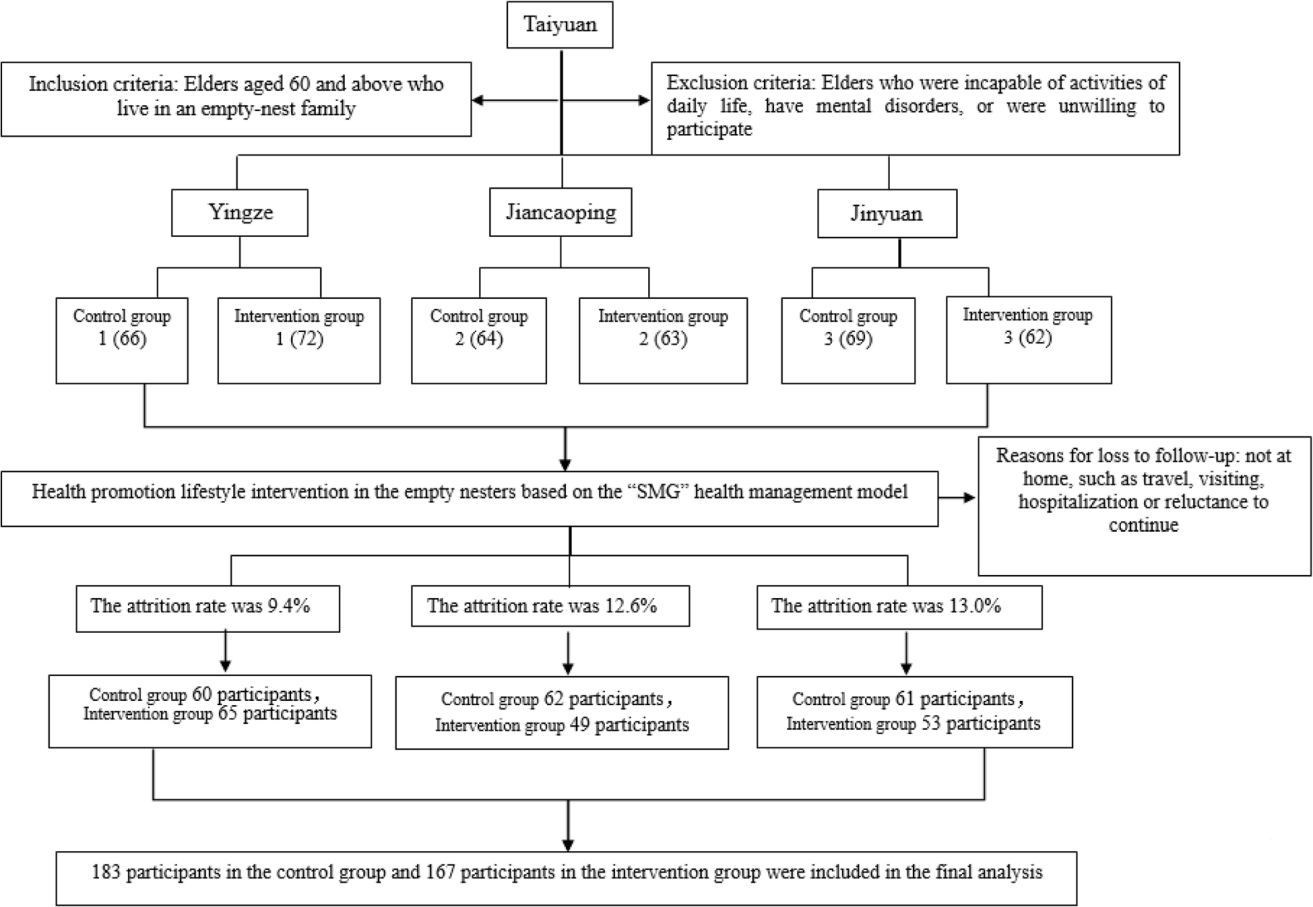
Flow chart of participant enrolment

The sample size was estimated according to the following formula ${\mathrm{N}}_1={\mathrm{N}}_2=\frac{2{\left({Z}_{\alpha }+{Z}_{\beta}\right)}^2\ {\sigma}^2}{d^2}$ . d: Mean_1_-Mean_2_ (Mean_1_, the mean of HPLP-C score among intervention group, Mean_2_, the mean of HPLP-C score among control group), σ: The standard deviation of control group. According to the pretest survey (Yingze), d = 6.9, σ = 14.14, α = 0.05, Z_α_ = 1.96, β = 0.1 Z_β_ = 1.282. The sample size was determined to be 88. Considering participant compliance and questionnaire effectiveness, we assumed that the missing rate is 10%, the sample size was expanded to 100. We used a stratified random cluster sampling method to sort six administrative districts of Taiyuan by economic level: Yingze District, Xiaodian District, Xinghualing District, Jiancaoping District, Wanbailin District, and Jinyuan District; the first two, middle two, and last two districts were, respectively, grouped into one region, resulting in a total of three clusters (high, medium, low economic levels). A third-party independent evaluator used the random-number table to select three districts from the three clusters, respectively, and then randomly selected a community from each of the selected districts using the same way. To guarantee the outcome of intervention, two residential areas were randomly selected from each community; one served as the intervention group, and one served as the control group, the interviewers were not blinded. All study procedures were approved by the Ethics Committee of Shanxi Medical university. If the older adults expressed interest in our intervention study, the researchers could contact and screen them according to the inclusion and exclusion criteria to determine if they were eligible to take part in the study. And the eligible participants were informed about the purpose and process of this study, and signed informed consent forms, and took part in the research group. The detailed inclusion and exclusion criteria and study process were depicted in Fig. 2(As shown in Fig. 2: Flow chart of participant enrolment).

This project has established a health-promoting lifestyles intervention team before the intervention. The team comprises of health management professionals, psychological counsellors, public nutritionists, social workers, and graduate student assistants, all of whom received lengthy and rigorous training of professional health intervention skills. The team employed popular social media platforms (QQ and WeChat) as the communication channel to facilitate coordination among team members and ease the transmission of health information and feedbacks. A designated staff recorded the information on The Empty Nester Health Promotion Lifestyle Record and maintained multimedia data. To prevent data contamination and minimize confounding factors, the interventionists were instructed to follow intervention measures as directed by the study staff and were monitored by the project managers.

### Measurements

We administered the questionnaire to the empty nesters at baseline and at follow-up respectively. The questionnaire was completed following an in-person interview between an interviewer and participants and was retrieved on the spot after the interviewer verified the information. The questionnaire consists of three sections:

1. Basic information: Basic information included an empty nester’s personal information, family circumstances, and community environment. This information relates to the demographic background of the empty nesters.
2. Health-Promoting Lifestyle Profile (HPLP-C) (Additional file 1): The Chinese version of the modified HPLP-C was developed and practiced in Taiwan [22]. We have adopted the HPLP-C to measure the lifestyle of empty nesters in six dimensions including self-realization, health responsibility, physical activity, nutrition, interpersonal relations, and stress management, with a total of 42 items. The score for each dimension was the summative score of its all items, and the HPLP-C score was the sum of the scores of all dimensions, ranging from 42 to 168. A higher score indicates better health lifestyle. The Cronbach’s α factor of the HPLP-C was 0.936, indicating a high-level internal reliability. Moreover, the factor loading ranges from 0.37 to 0.70, indicating good validity.
3. Empty Nester Health Promotion Lifestyle Record (Additional file 2): Designated study staff recorded the number of interventions conducted for each empty nester. At each follow-up, study staff recorded recent health and problems during the intervention of each empty nester.

### Intervention strategy

A previous cross-sectional lifestyle study [23] on 4901 empty nesters in Shanxi Province revealed that the HPLP-C score was 105.29 ± 19.68 (medium level). The nutrition mean score was 2.84, the highest among all dimensions, and the lowest mean score was 2.21 for health responsibility, suggesting that empty nesters were paying attention to nutrition. For nutrition, most high-score items were related to dietary habits, whereas the score for balanced nutrition was lower, indicating a need for targeting health education and that empty nesters needed to improve balanced nutrition and collocation of nutrition. Health responsibility referred to how much a person cared about his/her own health. This study revealed that the health responsibility score was the lowest among all dimensions, suggesting that empty nesters needed to pay more attention to regular physical examination, learn more about self-care, and actively seek the advice of health care workers and others. Furthermore, this study revealed that the physical activity score was low. Although many empty nesters were participating in physical activities, it was not scientific in the formulation of physical activity plan involving the arrangement of exercise intensity and the choice of exercise time for reaching good effects; particularly, the risk rating and safety testing for physical activity were unknown, which should be the key links during health management and behaviour intervention.

**Fig. 3:**
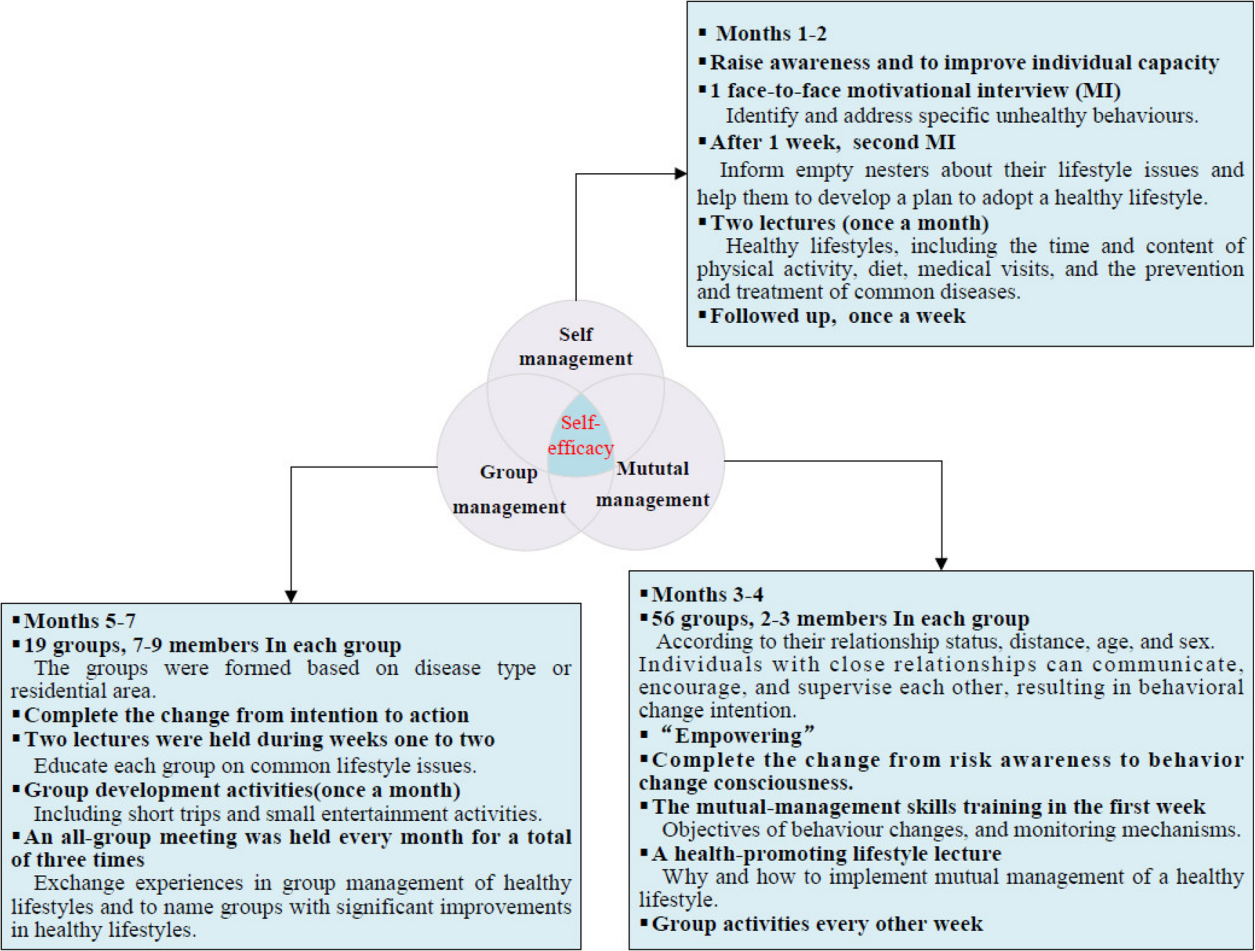
The detailed components of SMG intervention

We conducted our intervention study from October 2016 to May 2017. To maintain consistent intervention settings, we arranged the interventions in existing local health care centres or residential activity centres. The control group maintained their previous lifestyle without any additional measures imposed. The intervention group underwent the following phases of intervention: Self-management phase (2 months), Mutual-management phase (2 months), and Group management phase (3 months). During health management, the interventions focused on the unhealthy lifestyles of empty nesters. The intervention strategies were developed based on the “SMG” model, *Guidelines for the health of the Elderly in China* [24], *Chinese Dietary Guidelines for the Elderly in China* [25], and *the official website of China Healthy Lifestyle for all* [26]. The study staff in health management team guided health management and assisted in group assignment and implementation of the three-phase study. The Empty Nester Health Promotion Lifestyle Record was used to determine the number of interventions and the initiative of each empty nester. The detailed components are shown in Fig. 3. (As shown in Fig. 3: The detailed components of three-phase intervention).

### Statistical methods

SPSS v24.0 was used for data analysis. The chi-square test was used to compare the differences in basic characteristics among the intervention and control groups. Generalized estimating equations (GEE) models were used to assess the intervention effect over time on all the outcome variables, including the scores of HPLP-C, Self-realization, Health responsibility, Physical activity, Nutrition, Interpersonal relations and Stress management. In the GEE models, Time, Group and the interaction term between Time and Group (Time*Group) are independent variables. The coefficient of the interaction term Time*Group estimates the mean difference in the change of the outcome variables over time between the two treatment groups. Employment and education were controlled in the GEE models for all the outcome variables. Confidence level with *P* < 0.05 was considered statistically significant.

## Results

### Basic information

There were no significant socio-economic differences between the intervention group (*n* = 167) and control group (*n* = 183) with respect to gender, age, education, marital status, empty nester subtype, employment, monthly income, participation in social activities and chronic diseases among all three districts (As shown in Table 1). Three hundred and fifty elders completed the study and forty-six elders defaulted. There were significant differences between empty nesters who completed the study and those who defaulted with respect to Education (*χ*^*2*^ = 22.395, *P* < 0.001) and Monthly income (*χ*^*2*^ = 12.896, *P* = 0.005). There were 43 elders were primary education or below, and 3 were secondary education or above, and monthly income of 12 elders were 1000–3000 RMB, 10 elders were <  1000RMB and 24 elders were no incomes in the participants lost to follow-up.

**Table 1:** Comparison of the basic characteristics of the intervention and control groups

Characteristic	Category	Intervention group		Control group		*χ*^2^	*P*
		*n*	Proportion (%)	*n*	Proportion (%)		
Gender	Male	96	57.49	91	49.73	2.112	0.146
	Female	71	42.51	92	50.27		
Age	60–69 years	86	51.50	89	48.63	1.865	0.394
	70–79 years	59	35.33	76	41.53		
	80 years or above	22	13.17	18	9.84		
Education	primary education or below	83	49.70	106	47.90	2.377	0.134
	secondary education or above	84	50.30	77	52.30		
Marital Status	Married	113	67.66	131	71.58	0.848	0.838
	Never married	9	5.39	7	3.83		
	Divorced	5	3.00	5	2.73		
	Widowed	40	23.95	40	21.86		
Empty nester subtype	an elderly person of no family	23	13.77	20	10.93	1.018	0.601
	Relative empty nest	122	73.06	142	77.59		
	Absolute empty nest	22	13.17	21	11.48		
Employment	Yes	18	10.78	17	9.29	0.215	0.643
	No	149	89.22	166	90.71		
Monthly income	No income	51	30.54	61	33.33	1.922	0.589
	< 1000 RMB	43	25.75	45	24.59		
	1000–3000 RMB	40	23.95	50	27.32		
	> 3000 RMB	33	19.76	27	14.76		
Social activity participation	Most	19	11.38	18	9.83	1.419	0.701
	More	60	35.93	57	31.15		
	Less	69	41.31	85	46.45		
	No	19	11.38	23	12.57		
Chronic disease	Yes	44	26.35	65	35.52	3.425	0.064
	No	123	73.65	118	64.48		

### Intervention outcome

The analysis of Generalised estimating equations (GEE) models was performed to examine changes across time*group between the intervention and control conditions on scores of HPLP-C and six dimensions. Table 2 showed description statistics of pre-post mean scores and significance of time-group interaction. Moreover, we found significant differences after intervention associated with time and group interaction effects were in aspects of HPLP-C (mean score = 8.838, 95%CI:6.369–11.306), self-realization (mean score = 1.443, 95%CI:0.352–2.534), Health responsibility (mean score = 1.492, 95%CI:0.477–2.508), physical activity (mean score = 1.031, 95%CI:0.572, 1.491), nutrition (mean = 0.827, 95%CI:0.177–1.476), interpersonal relations (mean = 2.917, 95%CI:2.365–3.469) and stress management (mean score = 0.729, 95%CI:0.131–1.327).

**Table 2:** Generalised estimating equations results of HPLP-C and six dimensions scores between control and intervention group

	Baseline		Follow-up		Follow-up	
	Control (Mean, SD)	Intervention (Mean, SD)	Control (Mean, SD)	Intervention (Mean, SD)	Mean difference (95%CI)	*P*
N	183	167	183	167		
HPLP-C	107.34 (18.32)	106.93 (19.99)	106.96 (16.64)	115.39 (13.27)	8.838(6.369, 11.306)	< 0.001
Self-realization	37.30 (6.52)	37.03 (7.53)	36.98 (6.28)	38.16 (5.39)	1.443(0.352, 2.534)	0.01
Health responsibility	20.75 (4.99)	20.36 (5.50)	21.02 (4.53)	22.33 (4.68)	1.492(0.477, 2.508)	0.004
Physical activity	7.00 (2.20)	6.90 (2.19)	7.2 (2.01)	8.14 (2.08)	1.031(0.572, 1.491)	< 0.001
Nutrition	14.21 (2.74)	14.44 (2.76)	14.15 (3.47)	15.2 (2.17)	0.827(0.177, 1.476)	0.013
Interpersonal relations	12.65 (2.72)	12.82 (3.00)	12.54 (2.38)	15.62 (1.78)	2.917(2.365, 3.469)	< 0.001
Stress management	15.42 (3.03)	15.17 (3.43)	15.46 (2.76)	15.94 (2.71)	0.729(0.131, 1.327)	0.017

Model were adjusted for Education and Employment. The coefficient of the interaction term Time * Group estimates the mean difference in the change of the outcome variables over time between the two treatment groups

There was significant effect of education on HPLP-C score (Estimate = 8.833, *p* < 0.001), in that the elderly with secondary education or above had higher HPLP score than empty nesters with primary education or below. No significant effect of employment was found (See Table 3).

**Table 3:** Generalised estimating equations for HPLP-C score

Item	Estimate	CI	S.E.	Walls	*P*.
Time	−8.455	(−10.603, −6.307)	0.603	0.380	< 0.001
Group	−7.639	(−10.687, −4.592)	1.973	0.369	< 0.001
Time*Group	8.838	(6.369, 11.306)	1.260	49.23	< 0.001
Employment	4.150	(−2.151, 10.451)	3.215	1.666	0.197
Education	8.833	(6.369, 11.306)	1.670	27.99	< 0.001

The Estimate for Time is baseline to follow-up, for Group is control to intervention, for Employment is No compared to Yes and for Education is secondary education or above compared to primary education or below. Employment refers to current employment. In China, primary education or below refers to primary school education or no formal education. Secondary education or above refers to middle school education or above

## Discussion

In this study, we established special teams responsible for the intervention implementation and information collection in the community to guarantee the authenticity and credibility of the data. In addition, the questionnaire was examined to have good validity and reliability. Because of the good compliance of the participants, the data of original sample was complete, and all of the original data was used as the effective data analysed in the study. This study demonstrated that SMG-basedhealth-promoting intervention improved healthy lifestyles in empty nesters with satisfactory overall outcomes, indicating that the strategies played an instructive role in improving the lifestyles of empty nesters.

Traditional interventions on healthy lifestyles focused on the simple one-time health education or Single group which lead to the lack of active involvement and initiative for the participants, thereby presenting a limited short-term effect to the outcomes [27]. Such as a study used health-promoting educational intervention to improve lifestyles related to vaginal health among reproductive-aged women with vaginitis [28]. A community-based intervention study in rural Bangladesh examines the change in health-related quality of life (HRQoL) among (> or = 60 years) elderly persons as a result of health education intervention [29]. In contrast, the study of Lim LL found that team change, patient education, self-management, and improved patient-provider communication had the largest effect sizes to improve health care quality [30]. The “SMG” model is a multi-level integrated health management model of which the core is to elevate the self-efficacy, SMG-based interventions empower empty nesters to take ownership and participate in health management and enhance their confidence and determination for ongoing lifestyle changes through special education and group motivation activities. As a result, the health responsibility score was significantly improved after the intervention. In addition, the need to change lifestyle, self-manage, and adhere to lifelong treatment requires considerable commitment and self-discipline, especially when the disease is silent [30]. The “SMG” model focuses on mutual management and group management, thus embodying the “group (collective) effect” [31], which promotes individuals’ psychological and behavioural changes thanks to group monitoring and guidance and group interactions. For empty nesters, the group effect improves awareness and the capacity for mutual management in addition to self-management and enhances the role of group management in solving common health issues. The empty nesters were organized into groups based on disease types and residential areas to participate in customized intervention activities, which motivated the empty nesters to learn more about health management, help each other in the community, and form supportive social networks. As a result, the interpersonal relations score was also significantly improved. Moreover, mutual management and group intervention emphasized group activities, which to a certain extent encouraged empty nesters to leave the house, thereby contributing to significant improvements in the physical activity score. When related to time*group, the rest of the self-realization changed significantly. However, through the self-management from the “SMG” model, Self-identity and self-responsibility of empty nesters was improved. And based on mutual-management and group-management of the “SMG” model, participants showed an increase in social-identity, sense of daily satisfaction and expectations of future life, resulting in elevated self-realization. The use of social media platforms (QQ and WeChat) could enhance intervention including the ability for members of health-promoting lifestyles intervention team to share personal information that is aggregated and displayed to other members in real time [32]. But for the elderly, some of them don’t have social media platforms, so their information exchanges were based on face-to-face or paper communication. When related to time*group, the rest of the HPLP-C and six dimensions changed significantly.

This study revealed that the outcomes of SMG-based intervention were better in empty nesters with relatively high educational levels, indicating that education was an important facilitative factor for healthy behaviours among the older adults [13, 33]. Education level was related to how well the older adult accepted and understood new ideas and intervention compliance and thus was a factor for intervention outcomes. In a few cases, the HPLP-C score was lower after the intervention, which may come from measurement error. Inaccurate pre-intervention data may be the explanation for cases with decreased post-intervention scores. Some empty nesters did not fully comply with the instructions of this study, which, together with the characteristics of Chinese communities, indicated that community health service centres should play the role of evaluator for the intervention in future research to provide ongoing support for behaviour intervention among empty nesters.

The current study has several shortcomings that require further improvement and investigation. For example, the primary variables in this study were based on subjective questionnaires or scales, with no objective measures. The subjects may not have accurately responded to all questions because of the length of the questionnaire or scale and because of potential misunderstanding of the items, which may lead to bias and errors. Moreover, although measures were taken to minimize confounding factors, the effect could not be eliminated completely because the intervention was implemented through the daily life of empty nesters. Furthermore, the study period was short, and future research may extend the intervention period with multi-point measurement to evaluate the intervention process and outcomes. Solving the above-mentioned shortcomings will also provide valuable experience and directions for future research.

## Conclusions

“SMG” model is a multi-level integrated health management model of which the core is to elevate the self-efficacy and the aim is to improve health in empty nesters. In the “SMG” model, the whole intervention procedure was specifically divided into three stages, and participants were paired and grouped in terms of the disease types. This is the innovation of this model compared to other care models. The present study has yielded valuable information regarding the effectiveness of the “SMG” model for health-promoting lifestyles among empty nesters. Simultaneous interventions in three communities with different economic levels revealed that SMG-based lifestyle intervention effectively improved the lifestyles of empty nesters. The “SMG” model is one of the care models for empty nesters, which could improve their health-promoting lifestyles and should be heavily applied in the community in the future.

## Supplementary information

**Additional file 1.** Health-promoting lifestyle Profile-China.

**Additional file 2.** Empty Nester Health Promotion Lifestyle Record.

## Data Availability

Please contact author for data requests.

## Abbreviations

HPLP-C: Health-promoting lifestyle Profile-China
MI: Motivational interview
SMG: Self-management, Mutual management and Group management
