# Supplementary material for: The effectiveness of the “SMG” model for health-promoting lifestyles among empty nesters: a community intervention trial
Source: Health Qual Life Outcomes. 2019 Nov 8;17:168. doi: 10.1186/s12955-019-1222-x (PMC6839261; doi:10.1186/s12955-019-1222-x)
Supplement: Supplementary file 1 — Additional file 1. Health-promoting lifestyle Profile-China. [file 12955_2019_1222_MOESM1_ESM.doc]

**健康促进生活方式量表(HPLP-C)**

指导语：本问卷包含了您对目前的生活方式或个人习惯的叙述。以下共有42 个题目。每一个叙述都有4个可能的选择。请依据您自身情况选一个最能代表您个人生活习惯的答案，并在该方框内打“√”。

| 序号 | 项目 | 从不 | 偶尔 | 经常 | 总是 |
| --- | --- | --- | --- | --- | --- |
| 1 | 吃早餐 | 1 | 2 | 3 | 4 |
| 2 | 喜欢自己 | 1 | 2 | 3 | 4 |
| 3 | 每周做伸展运动至少三次（如扩胸运动、弯腰、踢腿等） | 1 | 2 | 3 | 4 |
| 4 | 每天花些时间放松自己 | 1 | 2 | 3 | 4 |
| 5 | 做身体检查或检验，并知道结果 | 1 | 2 | 3 | 4 |
| 6 | 对生命乐观、热诚 | 1 | 2 | 3 | 4 |
| 7 | 感觉自己正以积极的方式成长与变化中 | 1 | 2 | 3 | 4 |
| 8 | 与亲近的人讨论自己的问题及关心的事情 | 1 | 2 | 3 | 4 |
| 9 | 知道生活中压力的来源 | 1 | 2 | 3 | 4 |
| 10 | 感觉幸福、满足 | 1 | 2 | 3 | 4 |
| 11 | 每周激烈运动至少3次，每次持续20至30分钟（如爬楼梯、跳健身舞、快走等） | 1 | 2 | 3 | 4 |
| 12 | 每日三餐规律 | 1 | 2 | 3 | 4 |
| 13 | 阅读有关促进健康的文章或书籍 | 1 | 2 | 3 | 4 |
| 14 | 知道自己的长处和缺点 | 1 | 2 | 3 | 4 |
| 15 | 朝生命中长远的目标努力 | 1 | 2 | 3 | 4 |
| 16 | 赞赏他人的成就 | 1 | 2 | 3 | 4 |
| 17 | 当不同意医生的建议时，我会向他提出疑问，或征询另一位医生的看法 | 1 | 2 | 3 | 4 |
| 18 | 对未来充满期望 | 1 | 2 | 3 | 4 |
| 19 | 与亲近的人相互有身体的碰触（如搭背、牵手或更亲密的行为等） | 1 | 2 | 3 | 4 |
| 20 | 维持有意义的人际关系（指深层，非泛泛之交） | 1 | 2 | 3 | 4 |
| 21 | 食用富含纤维素的食物（如新鲜水果、蔬菜） | 1 | 2 | 3 | 4 |
| 22 | 每天花 15 至 20min 放松或冥想（静坐） | 1 | 2 | 3 | 4 |
| 23 | 与合格的专业人员讨论有关自己健康保健方面的事情 | 1 | 2 | 3 | 4 |
| 24 | 重视自己的成就 | 1 | 2 | 3 | 4 |
| 25 | 运动时测量自己的脉搏 | 1 | 2 | 3 | 4 |
| 26 | 花时间与亲密的朋友相处 | 1 | 2 | 3 | 4 |
| 27 | 测量血压，并知道自己的血压 | 1 | 2 | 3 | 4 |
| 28 | 参加有关改进我们居住环境的教育课程或活动 | 1 | 2 | 3 | 4 |
| 29 | 发觉每天都是充满乐趣及挑战的 | 1 | 2 | 3 | 4 |
| 30 | 每日吃含有五大类营养素的食物（包括蛋白质、脂肪、糖类、维生素及矿物质） | 1 | 2 | 3 | 4 |
| 31 | 睡觉前使自己全身肌肉放松 | 1 | 2 | 3 | 4 |
| 32 | 觉得自己的生活环境是舒适且令人满意的 | 1 | 2 | 3 | 4 |
| 33 | 从事休闲性的体能活动(如游泳、散步、踢足球、骑单车) | 1 | 2 | 3 | 4 |
| 34 | 就寝时，让自己想愉快的事情 | 1 | 2 | 3 | 4 |
| 35 | 以建设性的方式来表达自己的感受（指非批评、谩骂性，能提出具体建议） | 1 | 2 | 3 | 4 |
| 36 | 向健康专业人员咨询如何照顾好自己 | 1 | 2 | 3 | 4 |
| 37 | 每个月至少一次观察自己的身体有无异常症状或病征 | 1 | 2 | 3 | 4 |
| 38 | 我所设定的目标都是实际可行的 | 1 | 2 | 3 | 4 |
| 39 | 采用某些方法来减轻自己所面临的压力 | 1 | 2 | 3 | 4 |
| 40 | 参加有关个人健康保健方面的教育课程或活动 | 1 | 2 | 3 | 4 |
| 41 | 和我关心的人相互有身体的碰触（如搭背、牵手或更亲密的行为等） | 1 | 2 | 3 | 4 |
| 42 | 确信我的生命是有目的的 | 1 | 2 | 3 | 4 |
