# Supplementary material for: The effectiveness of the “SMG” model for health-promoting lifestyles among empty nesters: a community intervention trial
Source: Health Qual Life Outcomes. 2019 Nov 8;17:168. doi: 10.1186/s12955-019-1222-x (PMC6839261; doi:10.1186/s12955-019-1222-x)
Supplement: Supplementary file 2 — Additional file 2. Empty Nester Health Promotion Lifestyle Record. [file 12955_2019_1222_MOESM2_ESM.docx]

老年人健康促进生活方式记录表

Empty Nester Health Promotion Lifestyle Scale questionnaires

——For Self-management

记录员编号： 老年人编码：

（recorder number） (the elderly number)

| 活动名称lectures and follow up | 签到signature（√） |
| --- | --- |
|  |  |
|  |  |
|  |  |
|  |  |
|  |  |
|  |  |
|  |  |
|  |  |
|  |  |
|  |  |
|  |  |
|  |  |
|  |  |
|  |  |

| 个人存在的问题或其他（Personal problems） |
| --- |

老年人健康促进生活方式记录表

Empty Nester Health Promotion Lifestyle Scale questionnaires

——For Mutual-management

记录员编号： 互助组编码：

（recorder number） (mutual group number)

| 活动名称lectures and follow up | 签到signature (the elderly number) |
| --- | --- |
|  |  |
|  |  |
|  |  |
|  |  |
|  |  |
|  |  |
|  |  |
|  |  |
|  |  |
|  |  |
|  |  |
|  |  |

| 互助组存在的问题或其他（Problems of mutual group） |
| --- |

老年人健康促进生活方式记录表

Empty Nester Health Promotion Lifestyle Scale questionnaires

——For Group-management

记录员编号： 互助组编码：

（recorder number） (group number)

| 活动名称lectures and follow up | 签到signature (the elderly number) |
| --- | --- |
|  |  |
|  |  |
|  |  |
|  |  |
|  |  |
|  |  |
|  |  |
|  |  |
|  |  |
|  |  |
|  |  |
|  |  |

| 互助组存在的问题或其他（Problems of group） |
| --- |
